# Hypersensitivity and in-stent restenosis in coronary stent materials

**DOI:** 10.3389/fbioe.2022.1003322

**Published:** 2022-09-15

**Authors:** Wansong Hu, Jun Jiang

**Affiliations:** ^1^ Department of Heart Center, The Fourth Affiliated Hospital of Zhejiang University School of Medicine, Yiwu, Zhejiang, China; ^2^ Department of Cardiology, The Second Affiliated Hospital of Zhejiang University School of Medicine, Hangzhou, Zhejiang, China

**Keywords:** in-stent restenosis, hypersensitivity, drug-eluting stent, eosinophils, stainless steel

## Abstract

Coronary heart disease (CHD) is a type of cardiovascular disease with the highest mortality rate worldwide. Percutaneous transluminal coronary intervention (PCI) is the most effective method for treating CHD. However, in-stent restenosis (ISR), a long-term complication after PCI, affects the prognosis of patients with CHD. Previous studies have suggested that hypersensitivity reactions induced by metallic components may be one of the reasons of this complication. With the emergence of first- and second-generation drug-eluting stents (DES), the efficacy and prognosis of patients with CHD have greatly improved, and the incidence of ISR has gradually decreased to less than 10%. Nevertheless, DES components have been reported to induce hypersensitivity reactions, either individually or synergistically, and cause local inflammation and neointima formation, leading to long-term adverse cardiovascular events. In this article, we described the relationship between ISR and hypersensitivity from different perspectives, including its possible pathogenesis, and discussed their potential influencing factors and clinical significance.

## Introduction

Cardiovascular diseases (CVDs) are one of the leading causes of death worldwide. Globally, an estimated 18.6 million people died of CVDs in 2019. Since the 1970s, when the world’s first percutaneous transluminal coronary angioplasty (PTCA) was performed clinically, percutaneous coronary intervention (PCI) has become an important method of treating coronary heart disease (CHD). However, postoperative in-stent restenosis (ISR) and thrombosis are the main factors affecting its efficacy. ISR is defined as the presence of a stenosis greater than 50% of the diameter of the stent segment found on angiography that can clinically manifest as recurrent unstable angina pectoris, and in rare cases, acute myocardial infarction (Dangas et al., 2010). The incidence of ISR can be as high as 20–40%, and is caused by damage to the arterial wall due to various reasons, subsequently resulting in fibroproliferation, inflammatory response, and eventually neointimal hyperplasia (NIH) (Hoffmann et al., 1996). Few studies have shown that local inflammation caused by hypersensitivity to alloy components in metal stents may be one of the reasons for this complication (Table 1). Metal stents, which are foreign bodies, cause vascular remodeling, persistent endothelial dysfunction, and fibrin deposition via local inflammation (Torrado et al., 2018).

**TABLE 1:** Human studies and case reports implicating hypersensitivity in the process of restenosis.

Study	Model	Stent	Follow up	Results
Takashima et al	Human ISR specimens	PES in EES	4 months after PESs were employed n = 1	There are macrophages, foam cells, eosinophils, and persistent fibrin deposition surrounded the stent
Otsuka et al	ISR specimens	CYPHER stents	2 years after PCI n = 1	There are lymphocyte infiltration and hypersensitivity reaction with eosinophilia and proliferation of smooth muscle cells
Cihangir et al	Patch test	Cobalt chromium coronary stents	*n* = 61 (30 patients for ISR group; 31 as the control group)	7 of 31 patients (23%)
				In ISR group had nickel contact allergy (P <
				0.006)
Koster et al	Patch test and angiography	316L stainless-steel stents	6 months after PCI *n* = 131	All ten patients with positive patch Test results had restenosis (*p* = 0.03)
Kawano et al	ISR specimens and patch test	316L stainless-steel stent	Repeated ISR occurred 3 months after PCI *n* = 1	Histologically found the tissue is infiltrated with eosinophils, and patch test showed an allergic reaction to the stent
				material, including nickel and molybdenum
Rittersma et al	ISR specimens	316L stainless steel stents	*n* = 32 (16 patients had restenosis after balloon angioplasty, 16	In-stent restenotic tissue contained more smooth muscle cells (*p* < 0.001), anti-CD3 positive T cells (*p* < 0.001) and eosinophils (*p* = 0.012)
			patients had ISR)	
Saito et al	Patch test	316L stainless steel stents	*n* = 128	Nickel-positive was a significant predictor for CR-ISR(*p* = 0.0033)
			60 patients with the second ISR (study group) and 68 patients without the second ISR	
			(Control group)	
Granata et al	Patch test and OCT	ZES	6 months after PCI *n* = 1	In-stent restenosis was associated with nickel hypersensitivity
Niccoli et al	Basal ECP levels	BMS	24 months follow-up	ECP was associated with MACEs after BMS implantation
			*n* = 110	
HAJIZADEH et al	Peripheral blood eosinophil count and its percentage measured 6 weeks after DES implantation	DES	6 months follow-up *n* = 204	Eosinophil count of the peripheral blood was an independent predictor of ISR.
L.Pfoch et al	Skin prick and epi cutaneous testing with non-toxic paclitaxel dilutions	Taxus	2 weeks after PCI *n* = 1	There was a relation of the ISR and hypersensitivity reaction with a central role of paclitaxel
Jiasheng et al	ECP	DP-DES	*n* = 202	Serum ECP levels were higher in patients with ISR, is related to the reaction with coating drugs
		BP-DES		
Gabbasov et al	Blood plasma lev els of eosinophil cationic protein (ECP) and total immunoglobulin E (IgE)	Sirolimus-eluting stents	Follow-up angiography at 6–12 months *n* = 32	ECP was higher in patients with restenosis compared with that in patients without restenosis [17.7 ng/ml (11.2 -- 24.0) vs9.0 ng/ml (6.4 -- 12.9), *p* = 0.017]
SVEDMAN et al	Patch test	Au-stents and Ni-stents	*n* = 484	There is a correlation between contact allergy to gold, gold-stent, and restenosis (OR2.3, CI 1.0–5.1, *p* = 0.04)
Nakajima et al	Patch test	CoCr-EES and cobalt-chromium BMS	First time:6 months after PCI; Second time:2 weeks after PCI of ISR	Hypersensitivity to cobalt-chromium BMS led to acute progression of ISR.

ISR:in-stent restenosis; PCI: percutaneous intervention; ECP: eosinophil cationic protein; MACE: major adverse cardiac events.

CR-ISR: chronic refractory (CR) in-stent restenosis; OCT: optical coherence tomography.

**FIGURE 1:**
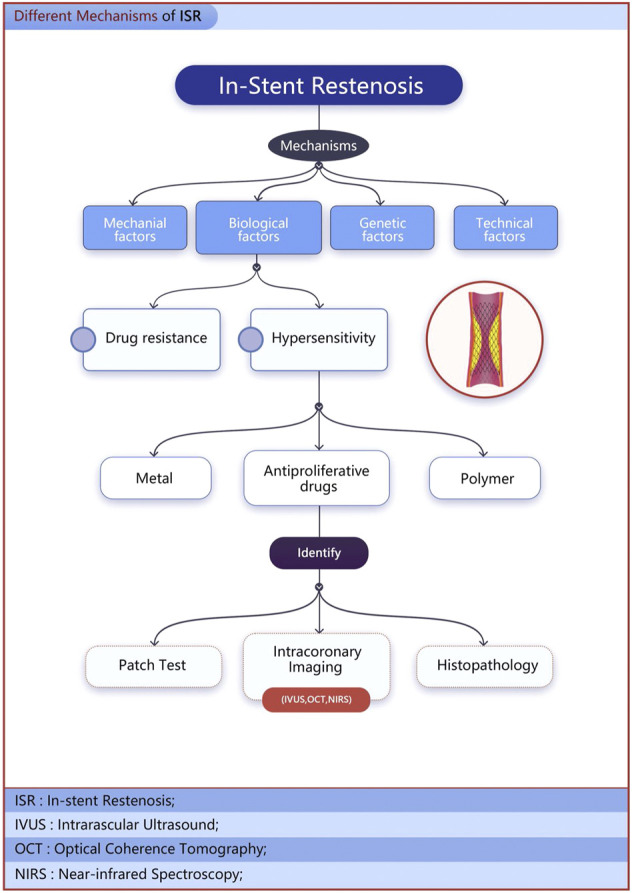
The underlying mechanisms of DES restenosis.

Over the past few decades, great strides have been made to reduce the incidence of ISR with the advent of drug-eluting stents (DES). Restenosis rates can be reduced by up to 10–20% in patients treated with first-generation DES. Nevertheless, according to incomplete statistics, the incidence of ISR still requires revascularization is 5 %–10%. The underlying mechanisms of DES restenosis can be considered to result from four factors: mechanical, biological, genetic, and technical factors (Aoki and Tanabe, 2021). In terms of biological factors, in addition to drug resistance, allergic inflammatory responses to polymer and metal scaffold platforms cannot be ignored (Figure 1) (Giustino et al., 2022).

In this review, we explored the relationship between hypersensitivity and the different components of different stents, including their potential mechanisms for promoting restenosis and their impact on prognosis. In addition, we summarized the evidence that ISR is associated with allergic reactions and discussed its current diagnosis, treatment strategies, and future prospects.

## Inflammatory mechanism of restenosis

Inflammatory response is one of the pathogenic mechanisms leading to adverse events after stent implantation, and its role in the occurrence and development of ISR after PCI has been confirmed in several studies (Welt and Rogers, 2002). Balloon expansion and stent implantation can cause vascular endothelial injury to varying degrees, and pro-inflammatory factors, such as tumor necrosis factor (TNF), interleukin-1, and interleukin-6 released at the injury site, mediate the adhesion and aggregation of inflammatory cells. The inflammatory process can stimulate proliferation, differentiation, and extracellular matrix synthesis of vascular smooth muscle, eventually leading to the proliferation of local neointima (Kornowski et al., 1998). In addition, damaged vascular endothelium is prone to platelet and fibrin deposition, which is associated with the formation of mural thrombi (Byrne et al., 2015).

Classical and allergic inflammation are the two main inflammatory response mechanisms. The activation pathway of the former involves monocytes, macrophages, neutrophils, and T lymphocytes, whereas, the latter is mainly caused by eosinophils and mast cells (Montone et al., 2013).

In the classical inflammatory response, C-reactive protein (CRP) is a common inflammatory marker. Few studies have found that plasma CRP levels are significantly increased in patients with restenosis. Clinically, it can be used as an independent predictor of major adverse cardiac events (MACE) and restenosis after stent implantation (Buffon et al., 1999). Other inflammatory markers, such as plasminogen activator inhibitor (PAI-1) and matrix metalloproteinases (MMPs), are also considered to be associated with first-generation DES restenosis (Katsaros et al., 2008; Katsaros et al., 2010).

Eosinophils and mast cells are effector cells involved in allergic inflammatory responses (Costa et al., 1997). Eosinophils can secrete growth factors, chemokines, and interleukin-1 (Fulkerson and Rothenberg, 2013). Mast cells are involved in the release of histamine, tryptase, chymase, a series of cytokines and chemokines, platelet activating factors, and arachidonic acid products (Shi et al., 2015). Cellular mediators of allergic inflammation also play roles in the formation and development of coronary plaques. Among them, histamine and leukotrienes are effective vasoactive inflammatory molecules that can increase the vascular permeability and activate endothelial cells (Kunder et al., 2011). Tryptase and chymase may be involved in the degradation of high-density lipoprotein (HDL) (Lee et al., 2002), which may be related to plaque rupture in patients with acute coronary syndrome (Lee-Rueckert and Kovanen, 2006). Additionally, mast cells promote the activation of T cells and macrophages, leading to endothelial cell proliferation and fibrosis. In fact, both the types of cells are functionally capable of regulating each other, forming the so-called “allergic effector units” (Gangwar et al., 2016).

## Hypersensitivity of coronary stent materials

### Hypersensitivity to metal components

Most coronary stents, especially bare metal stents (BMS) and first-generation DES, are made of 316 L stainless steel, which contains metal elements such as nickel, chromium, and molybdenum. These metal ions are eluted from the stent by the blood, saline, proteins, and mechanical stress. It is estimated that up to 17% of the population is allergic to nickel and another 1–2% of the population is allergic to other elements, such as cobalt and chromium, to varying degrees (Basko-Plluska et al., 2011). Allergies related to metal implants are well documented in orthopedics, dentistry, and other fields (Honari et al., 2008). Metal ions eluted from the device may bind to endogenous proteins in complexes or directly activate T cell receptors in a super-antigen fashion. Additionally, they may lead to macrophage activation and cause delayed allergic reactions. The allergic reactions may be localized as local dermatitis, systemic as eczema, or may induce a syndrome similar to systemic lupus erythematosus. Hypersensitivity to related metal components has been found in patients with late adverse cardiovascular events (Granata et al., 2015) such as ISR and late stent thrombosis (LST) (Kataoka et al., 2012).

Köster et al. were the first to demonstrate that patients with delayed hypersensitivity reactions to metals, especially nickel, have a higher incidence of ISR(Köster et al., 2000). However, due to the limitations of retrospective studies, only 8% of patients in the study had positive results. Compared with the high incidence in the population, this finding is not surprising. In addition, investigators have pointed out that patients without suspected restenosis were not investigated in this study; hence, it is difficult to rule out possible metal allergy, which is an important missing control group (Keane et al., 2001). Some follow-up studies of ISR in patients treated with stainless steel stents did not confirm Köster’s original observations. Furthermore, Saito et al. found that tissue response to nickel is a major factor in chronic refractory ISR (Saito et al., 2009). New-generation DES use metal alloys, such as cobalt-chromium (CoCr) or platinum-chromium (PTCR), which, in contrast, have thinner struts and lower nickel content than stainless steel stents (Gori et al., 2019). However, nickel allergy remained significantly associated with ISR in CoCr stent-treated patients (Aliagaoglu et al., 2012). Additionally, a study reported local hypersensitivity and restenosis at the site of stent fracture after CoCr stent implantation, suggesting a potential role of the accelerated release of metal ions (Mori et al., 2017).

Previous case reports have demonstrated repeated incidence of ISR after receiving CoCr stents in patients with proven nickel allergy. After switching to bio-absorbable vascular stents (Jurado-Roman et al., 2017) and prednisolone anti-allergic treatment (Nakajima et al., 2016) after PCI, the patients’ prognosis significantly improved. In 2016, Nakajima also reported a case of recurrent ISR and overt metal allergy after implantation of CoCr everolimus-eluting stents (CoCr—EES), in which recurrent restenosis was terminated with prednisolone and tranilast treatment (Nakajima et al., 2016).

Studies have shown that nickel salts such as nickel chloride can directly activate vascular endothelial cells and upregulate the expression of intercellular adhesion molecule 1 (ICAM-1) (Messer et al., 2005). As a cell surface glycoprotein and adhesion receptor (Hubbard and Rothlein, 2000), ICAM-1 plays a role in leukocyte transendothelial migration (TEM). In response to inflammatory stimuli, ICAM-1 is involved in the regulation of leukocyte rolling and adhesion interactions with the vessel wall in the vascular endothelium and guides leukocytes across the endothelial layer (Yang et al., 2005), thereby promoting the occurrence of local in-stent restenosis. Additionally, ICAM-1 is involved in many other physiological processes, including immune cell effector functions, clearance of pathogens and dead cells, and activation of T cells. Related studies have also been conducted on the development, metastasis, and prognosis of tumors. Furthermore, studies have demonstrated that ICAM-1 expression can be induced in conjunctival epithelial cells (ECs) of allergic patients, revealing its role as a marker of allergic inflammation (Bui et al., 2020).

However, there have always been a controversy regarding the allergy and restenosis associated with stainless steel stents. Few studies do not report a link between these factors (Thyssen et al., 2011; Slodownik et al., 2018). In a prospective study, Norgaz et al. did not find an association between nickel allergy and the development of ISR in patients with stainless steel stents (Norgaz et al., 2005). Similarly, Iijima et al. prospectively assessed the differential relationship between metal allergy in initial ISR and after restenosis treatment and found that metal allergy was not associated with restenosis after the initial stent implantation but was associated with recurrent restenosis (Iijima et al., 2005). It is worth noting that unlike the patch test method in previous studies, Santiago et al. only included patients with a history of metal allergy before coronary stent placement by querying personal history. The possibility of sensitization directly caused by the stent placement was ruled out. The findings showed that a history of metal allergy was not associated with adverse outcomes in patients undergoing PCI (Romero-Brufau et al., 2012). Furthermore, in a retrospective study, Svedman et al. found that gold-plated stents were significantly associated with gold allergy and restenosis. Conversely, this behavior was not observed for nickel in this study. These findings suggest that gold is a stronger sensitizer than nickel and may elicit a stronger immune response leading to endothelial cell proliferation (Svedman et al., 2009). Nevertheless, larger prospective studies and randomized controlled trials are needed to confirm the association between this metal allergy and ISR.

### Hypersensitivity to antiproliferative drugs in DES

The allergic reaction resulting from the use of DES is not solely from the non-drug components of the stent. Compared with BMS, DES incorporate antiproliferative agents to prevent restenosis. There are two main classes of impregnated drugs in first-generation DES: inhibitors of the mammalian target of rapamycin (mTOR) (e.g., sirolimus and its analogs) and paclitaxel and its derivatives. The first drug used in the Cypher stents was sirolimus. Sirolimus (rapamycin) is a macrolide antibiotic extracted from S. hygroscopicus (Aminian et al., 2009). It is a multifunctional serine-threonine kinase that acts on IL-2-mediated signal transduction pathways, and is a central regulator of cell growth, proliferation, and apoptosis. Therefore, it is used in immunosuppressive therapy for cancer and as an anti-rejection agent after transplantation. Moreover, it is used in coronary stents to reduce the neointimal formation and restenosis (Kawano et al., 2004; Steffel and Tanner, 2007). Although sirolimus is mostly considered to be an unlikely cause of allergy as it generally reduces eosinophil infiltration and histamine release, clinical adverse reactions to sirolimus such as bone marrow suppression, hyperlipidemia, and hypercholesterolemia, are still visible (Brara et al., 2003). In addition, life-threatening coronary spasms have been reported, which may be related to severe endothelial dysfunction (Wheatcroft et al., 2006). This is similar to the clinical complications of the type I variant of Kounis syndrome. At the same time, rapamycin was also found to increase the expression and activity of thrombin and tumor necrosis factor-α-induced endothelial tissue factor (Steffel et al., 2005).

In an animal study, histopathological examination of the heart of laboratory rats administered with sirolimus revealed a focal myocardial infarction (Walpoth and Hess, 2004). The incidence was positively correlated with the drug dose. In addition, animal experiments have shown that a rat model of synthetic vascular grafts treated with systemic or topical rapamycin has a propensity for thrombosis (Walpoth et al., 2001). Previous human studies have also reported that allergic vasculitis after DES implantation may be associated with late and very late stent thrombosis, and occurs almost exclusively with first-generation sirolimus-eluting stents (SES, Cypher) (Cook et al., 2009). In addition, Nakazawa et al. evaluated patients’ coronary stent histomorphology and found that although SES can better inhibit NIH, the allergic inflammation mediated by cells including eosinophils and lymphocytes is more serious and is the main cause of LST compared with paclitaxel-eluting stents (PES) (Nakazawa et al., 2008). In contrast, LST in PES is mostly secondary to the malpositioning caused by excessive fibrin deposition.

Paclitaxel was isolated from the bark of Taxus japonica and was later used as an anti-restenosis drug for the TAXUS stent. Unlike mTOR inhibitors, paclitaxel is a cytotoxic agent that binds to *β*-tubulin and causes cell cycle arrest in the second growth phase by inhibiting the microtubule assembly (M phase of the cell cycle), leading to the dissolution of mitotic spindle structures (G2) and mitosis (M) (Blagosklonny et al., 2004)and inhibiting the proliferation of human endothelial cells. Allergic reactions such as neutropenia, thrombocytopenia, gastrointestinal symptoms, and peripheral neuropathy are common in patients with various cancers treated with paclitaxel (Picard, 2017). Although it is an effective antirestenosis drug, its safety has been questioned. A previous study found that paclitaxel may be an important cause of the excessive deposition of intravascular fibrin, which is related to LST (Nakazawa et al., 2011). Pfoch also reported a case of anaphylaxis 2 weeks after PES stent placement in a patient who was desensitized after antihistamine therapy (Pfoch et al., 2009). Owing to the timely detection and treatment, the patient did not develop ISR during the follow-up period of more than 1 year. However, it is worth noting that it is often easy to ignore the follow-up data in patients whose early allergic reactions are not obvious in the whole body and could possibly turn fatal. Although DES have been shown to reduce restenosis rates, allergic reactions to stent components have the opposite effect.

Compared with first-generation DES, second-generation DES use derivatives of sirolimus, such as Evorolimus and Zotarolimus, as carrier drugs. Compared with sirolimus, they can be used at lower drug concentrations and have reduced toxicity compared. However, Otsuka et al. reported a case of ZES and EES implantation. The patient had three ZES and one EES implanted for severe LAD lesions and died 238 days later. Histological examination of the scaffold revealed persistent inflammation and fibrin deposition with marked infiltration of eosinophils, T-lymphocytes, and multinucleated giant cells (Otsuka et al., 2015). Moreover, hypersensitivity pneumonitis has been previously reported in kidney transplant patients treated with sirolimus (Shin et al., 2013). Although ZES-related allergic events are rare, the biological toxicity of zotarolimus cannot be ruled out (Takashima et al., 2015).

### Hypersensitivity to polymer in DES

Polymers, an important part of DES, can control the release of anti-proliferative drugs to ensure the anti-restenosis efficacy. As carriers for topical administration, polymers in first-generation DES such as polystyrene-b-isobutylene-b-styrene of the Taxus Express PES, Cipher sirolimus polyethylene vinyl acetate (PEV-A), and polybutyl methacrylate (PBMA) of SES-eluting stents can effectively control drug release and significantly reduce the rate of restenosis (Byrne et al., 2017). However, data suggest that polymers used in first-generation DES have poor biocompatibility and are associated with late clinical adverse events. Allergic reactions have been reported with the use of polymers, such as those in latex and vinyl gloves. These allergic reactions are usually type IV hypersensitivity reactions caused by low-molecular-weight compounds called haptens. In 2004, a case of a local allergic reaction to Cipher SES was reported, possibly triggered by its polymers (Virmani et al., 2004b). Notably, the local inflammatory response was found to be more pronounced after 90 days, when the antiproliferative drugs were released. In addition, an animal experiment found localized extensive inflammation with abundant eosinophils at 28 and 90 days in Cipher stents implanted in porcine coronary arteries (Virmani et al., 2004a). However, in polymer-free metal stents, the inflammation was less pronounced at 90 days than at 28 days, and the inflammatory response was found to be polymer-related at longer durations. Similar reports have been reported in other clinical trials. These results suggest that polymers are associated with excessive inflammation and stent thrombosis (ST). In addition, it has been reported that the polyethylene-vinyl acetate compound of Cipher copolymer can cause an inflammatory response in 25% of rabbits when used as an antigen delivery matrix (Niemi et al., 1985). Both PES and SES elicited distinct inflammatory responses in animal models for over 90 days. The second-generation DES can reduce the inflammatory or allergic reaction caused by polymers by improving the biocompatibility of the stent polymer coating or by applying a degradable polymer coating. Second-generation DES use highly fluorinated polymers or amphiphilic polymers (i.e., PVDF-HFP and BioLinx) (Strohbach and Busch, 2015), which reduce the platelet adhesion and activation compared to non-fluoropolymer-coated metal stents and have better resistance than previous polymers (Torii et al., 2020). However, a case report found persistent fibrin deposition and extensive periarthritis in stent segments at autopsy 8 months after implantation of the CoCr-EES and Resolute zotarolimus-eluting stent (R-ZES), caused by palisade macrophage cells, T lymphocytes, eosinophils, and multinucleated giant cells, suggesting the occurrence of allergic reactions (Otsuka et al., 2015). This can be attributed to either of the two components of DES: the drug and the polymer. The coexistence of PBMA as a component of the polymer in both the scaffolds should be suspected. In addition, many new durable and biodegradable polymers (Gong et al., 2010) have been studied. Most biodegradable polymers are synthetic polyesters of the polylactic acid-hydroxy acid family. Studies have found that they decrease MACE within 24 months and show a certain advantage (Han et al., 2008). However, more clinical studies are needed to determine their long-term prognosis. Furthermore, it has been suggested that neither the polymer nor the drug could be an allergen, since allergic ISR still occurs 8 and 12 months after stent implantation in the absence of both biodegradable polymer and sirolimus (Jimba et al., 2020).

## Kounis syndrome and allergic inflammation

The prognosis of patients with acute coronary lesions undergoing PCI is confirmed to be related to the activation of various inflammatory cells and the production and release of several mediators (Niccoli et al., 2010b).

Previously, researchers defined an allergy-related acute coronary syndrome as “Kounis Syndrome” (KS). The pathophysiology of KS is characterized by a localized allergic response leading to mast cell activation and release of inflammatory mediators. When this process exceeds a certain threshold, coronary spasm and plaque erosion or rupture can occur (Kounis et al., 2007). There are three variants of KS. The type I variant is the most common, accounting for approximately 72.6% of the cases, with a clinical manifestation in the form of coronary artery spasm with or without an increase in myocardial enzyme and troponin levels. The type II variant (22.3%) is accompanied by plaque erosion or rupture, manifesting as acute myocardial infarction. Type III variant (5.1%) involves coronary stent thrombosis due to allergic reactions (Kounis et al., 2012; Abdelghany et al., 2017). KS, a possible manifestation of hypersensitivity to stent components, may play a key role in the development of acute or late thrombus formation in DES (Chen et al., 2009).

ISR is a multifactorial and complex process, and studies have suggested that inflammation and immune imbalance play important roles. Eosinophils are important effector cells associated with allergy and play an important role in promoting allergic inflammation by releasing pro-inflammatory mediators (histamine and leukotriene C4) (Bochner, 2000). Eosinophils have been demonstrated to be reliable predictors of ISR after DES implantation (Hajizadeh et al., 2017). Histopathological studies have shown that eosinophils are associated with ISR with BMS. Numerous pathological studies have also shown inflammatory responses such as macrophages, foam cells, eosinophils, and persistent fibrin deposition around the scaffold in patients with ISR. In particular, the infiltration of a large number of eosinophils suggests hypersensitivity (Rittersma et al., 2006). Similarly, some case reports have revealed histopathological findings of lymphocytic and eosinophilic infiltration following Cipher stent implantation. Eosinophils may accumulate late and secrete inflammatory factors, initiate inflammatory and hypersensitivity reactions, and exacerbate tissue damage, thereby promoting smooth muscle cell migration and proliferation, leading to lumen narrowing and the occurrence and development of ISR (Gabbasov et al., 2009). Notably, eosinophils are equally important in the promotion of thrombosis (Sakai et al., 2009). Eosinophils also synthesize and release many other pro-angiogenic cytokines such as IL-8, IL-6, transforming growth factor-beta, and granulocyte-macrophage colony-stimulating factor (GM-CSF), both of which play a role in promoting wound healing and maintaining allergic inflammation (Munitz and Levi-Schaffer, 2004). Nevertheless, most related studies are single case reports or small sample clinical observations. There is a lack of clinical studies with larger samples for verification.

The eosinophil cationic protein (ECP) is a sensitive marker of eosinophil activation (Niccoli et al., 2010a; Niccoli et al., 2014a), and a variety of biological activities that interact with other immune cells and plasma proteins. Elevated eosinophil activation plays an important role in the pathogenesis of restenosis in DES (Gabbasov et al., 2011). ECP also upregulates the ICAM-1 expression in endothelial cells, allowing monocytes to adhere to endothelial cells, which is thought to be an essential step in atherogenesis. In addition to its pronounced cytotoxic activity, ECP has several regulatory activities *in vitro*, including the inhibition of proliferating T lymphocyte responses to antigens and inhibition of B lymphocyte synthesis of immunoglobulins. In a prospective study, baseline serum ECP levels were used to predict the risk of MACEs after BMS and first-generation DES implantation (Niccoli et al., 2009; Niccoli et al., 2011). In addition, Niccoli et al. found that unlike CRP, ECP is associated with the severity of coronary atherosclerosis (Niccoli et al., 2014b). Moreover, this study found that serum ECP levels were significantly elevated in patients with advanced ISR and were an independent risk factor for ISR.

## Diagnosis and evaluation

Coronary angiography remains the clinical standard for diagnosing ISR and assessing its severity. Different morphologies indicated by angiographic results are of great significance for the classification, treatment, and prognostic evaluation of ISR. However, coronary angiography has certain limitations, especially in assessing the lumen size and plaque characteristics. Intracoronary imaging techniques allow for a detailed, objective assessment of the extent and morphology of lesions by changing the imaging modality. Furthermore, it has become an important tool for understanding the pathophysiology associated with ST and ISR(Koskinas et al., 2016; Mintz and Guagliumi, 2017).

Current commonly used intracoronary imaging tools include intravascular ultrasound (IVUS) and optical coherence tomography (OCT). IVUS has the ability to visualize the coronary lumen and vessel wall and can help delineate the outer elastic lamina behind stents, thereby revealing the actual vessel size, assessing the post-stent under-expansion, and distribution of NIH. A classification system for DES-ISR was proposed based on the inspection findings under IVUS (Kang et al., 2011). In contrast, OCT provides higher-resolution imaging that can better characterize the tissue, delineate the lumen-intima interface, and determine the distribution of stent struts. Additionally, it can visualize the macrophage clumps and vascular components and distinguish the white thrombi from the red thrombi. It is important to measure the fibrous caps and identify unstable plaques (Kashiwagi et al., 2013; Taruya et al., 2015). The ISRs of BMS and DES exhibit different characteristics in OCT. The BMS-ISR typically shows a homogeneous hyperintense tissue band on OCT, reflecting its NIH richness in smooth muscle cells. In contrast, DES-ISR is seen as unevenly distributed on OCT, suggesting that the neointima contains more proteoglycans or fibrin, and fewer cells. In addition, OCT is helpful for the assessment of neoatherosclerosis. A new classification scheme was recently proposed to describe the mechanism of ISR by using OCT to guide the associated treatment (Gonzalo et al., 2009). Near-infrared spectroscopy is another less-used imaging technique capable of localizing and quantifying lipid core load (Roleder et al., 2017); however, its clinical benefit is currently limited to relevant case reports.

Intracoronary imaging can be helpful in the detection of NIH in the context of allergic reactions; however, the clinical diagnosis of allergic reactions is mostly derived from the histopathology of restenotic stents or autopsy pathology. In addition, serum eosinophil level is a sensitive marker of allergic reactions. The detection of allergy to metal components mostly adopts a unified patch test, and the standard of positive reaction is an inflammatory reaction after 48 h or 72 h, accompanied by erythema, edema, papules, or other infiltrative changes (Johansen et al., 2015).

## Drug therapies

Repeated DES implantation is the most effective treatment for ISR (Giacoppo et al., 2015; Siontis et al., 2015); however, interventional procedures are subject to complications such as perioperative myocardial injury (PMI) (Nano et al., 2022). In addition to the reopening of diseased blood vessels, drug therapy for allergic inflammation is worthy of further study. Related drugs mainly inhibit the activation and local recruitment of allergic inflammatory effector cells and release of allergic response mediators.

Statins are widely used in the treatment of CVDs and have anti-inflammatory, antioxidant, and anti-atherosclerotic functions. In addition, statins exhibit immunomodulatory effects. Fluvastatin is a potent inhibitor of IgE-mediated activation and degranulation of basophils and mast cells (Kolawole et al., 2016).

Leukotrienes mediate various inflammatory and allergic responses and are produced by the metabolism of arachidonic acid via the 5-lipoxygenase pathway. Leukotriene B4, produced by enzymatic hydrolysis, can induce chemotaxis and adhesion of inflammatory cells such as neutrophils and macrophages to vascular endothelial cells (De Caterina and Zampolli, 2004). Leukotriene receptor antagonists are widely used as anti-inflammatory and anti-allergic drugs. In a study of the low-density lipoprotein (LDL) receptor mouse model, it was found that the degree of arterial injury in mice with 5-lipoxygenase gene deficiency was significantly reduced, suggesting that 5-lipoxygenase may play an important role in atherosclerosis (De Caterina and Zampolli, 2004). Similarly, a clinical study of patients with acute coronary syndromes found significant reductions in the volume of noncalcified coronary plaques in patients treated with inhibitors of 5-lipoxygenase activity (Tardif et al., 2010).

Moreover, the traditional mast cell stabilizer, sodium cromoglycate, has a good therapeutic effect on allergic reactions as it inhibits the release of allergic response mediators such as histamine and serotonin by stabilizing the mast cell membranes and preventing degranulation. The mas-related G protein-coupled receptor-X2 (MRGPRX2) receptor is thought to be one of the possible links between cardiovascular events and allergies (Azimi and Lerner, 2017). Novel mast cell stabilizers such as QWF (Boc-Gln-D-Trp [Formyl]-Phe benzyl ester tri-fluoroacetate) inhibit the substance P-induced mast cell degranulation and inflammatory responses by antagonizing MRGPRX2. The study also found that in LDL receptor-deficient (Ldlr−/−) mice, mast cell stabilization may have played an important role in delaying the progression of coronary atherosclerosis, reducing inflammation, and improving lipid metabolism (Wang et al., 2013).

## Future directions

The main mechanism for the occurrence of adverse reactions after DES implantation is the activation of both the classical and allergic inflammatory pathways. All DES components, including metal and polymer coatings, can induce hypersensitivity reactions individually or synergistically. In addition to acute or late ST, the IRS due to hypersensitivity-induced NIH also significantly affects patient prognosis. Regarding metal allergy, some researchers have proposed the use of nickel-free stainless steel materials and degradable metal stents. Recently, a study has considered the use of new titanium-alloy stents. Titanium alloy as a drug storage layer can replace the original polymer coating to overcome the possible existing polymer sensitization by forming a nanotube-like oxide layer on the anodized surface (Soliman et al., 2019). The study showed that in patients with acute coronary syndromes, CoCr-titanium-coated stents were non-inferior to the platinum-chromium biodegradable polymer, EES, in inducing major cardiac events at 12 months (Tonino et al., 2020). Despite the biocompatibility of the target moiety and improved tissue specificity and cellular uptake (Yin et al., 2014), nanoparticles, as a new generation of smart drug delivery materials, still require extensive research to evaluate their safety issues (Cherian et al., 2021). In addition, clinical evaluation of polymer-free DES is also underway. In a clinical follow-up trial of up to 3 years, polymer-free amphilimus-eluting stents were compared with the new-generation permanent-polymer zotarolimus-eluting stents in 1–3 years of TLF (in terms of target lesion failure) (van Hemert et al., 2021). No significant differences were observed in studies with longer follow-up periods (Kufner et al., 2020). Among the many factors that contribute to ISR, implantation is usually the most important and relatively controllable factor (Farooq et al., 2011).

With the gradual deepening of the concept of “intervention without implantation,” fully bioabsorbable stents should have gradually entered the clinic. The advantages of bioresorbable coronary scaffolds (BRS) can be divided into the following aspects: 1) The absence of permanent stent implants can restore the response of blood vessels to normal physiological stimuli, which is helpful for the dilatative remodeling of blood vessels at the late stage; 2) Without metal stents, continuous stimulation of the material can reduce the occurrence of local chronic inflammatory reactions, thereby reducing intimal hyperplasia and thrombotic events; 3) BRS will not affect revascularization after complete absorption, nor will it affect the noninvasive imaging. Related absorbable magnesium alloy stents have been studied and have been shown to demonstrate better antithrombotic properties in clinical trials (Sakamoto et al., 2018). However, long-term follow-up data are required to verify its safety and efficacy.

Stent coverings for nitric oxide donors and stents embedded with anti-inflammatory and anti-allergic drugs are gradually being developed (Kural et al., 2019). However, whether these can address this worrying complication remains a question worthy of further clinical research. Scientists are actively seeking new interventions to reduce the inflammation after stent implantation. Microribonucleic acids are a class of small non-coding RNAs that play important roles in the initiation and resolution of inflammation after vascular injury. The MiR-21 stem loop plays an important role in the activation of smooth muscle cells (SMCs) and macrophages after vascular injury. Animal studies have shown that the genetic ablation of the miR-21 stem loop reduces neointimal formation following stent implantation in mice (McDonald et al., 2015).

## Summary

ISR remains a challenging problem in the cardiovascular field and its occurrence is often multifactorial, where local inflammation leading to aggressive neointimal proliferation and advanced neoatherosclerosis is common. Stratification according to the etiology and pathogenesis of ISR may be necessary to guide the individualized treatment (Shlofmitz et al., 2019). Stent-related allergic reactions are commonly reported with the use of BMS and first-generation DES. Although the use of second-generation DES has improved in-stent platform design and antiproliferative drugs, polymer coatings and allergic reactions related to stent components have been previously reported. In addition, DES-related hypersensitivity is associated with ST (Yokouchi et al., 2010) and late-acquired stent dislocation. Hypersensitivity-related mediators are involved in platelet activation, and endothelial dysfunction caused by inflammatory responses that may induce neoatherosclerosis, thereby promoting ST (Chioncel et al., 2021). Compared with BMS, late lumen loss in DES occurred more than 9 months later and increased gradually, a phenomenon known as late catch-up. A meta-analysis showed that allergy to stent materials significantly increased the risk of ISR (Gong et al., 2013). However, the limitations of the meta-analysis are that the included studies were case-control studies and not a single prospective study was included.

Nevertheless, physicians should be aware of the occurrence of allergic reactions associated with stent placement. Further research is required to improve the biocompatibility of coronary stents. Clinically, high-risk individuals with potential allergic reactions to stent components after DES implantation can be identified based on the evaluation of markers, such as eosinophil count and ECP. In addition, there is a need to develop new and reliable diagnostic methods for identifying the potential allergens. The predictive value of related indicators for poor prognosis, such as ISR, can also help optimize the clinical management of patients. In patients with potential allergic reactions, patch testing before and after stent placement and subsequent risk stratification for allergic predisposition may be necessary, while few patients may require combined anti-allergic therapy. Furthermore, given the development of anaphylaxis and poor prognosis of ISR, more research is needed to understand the specific pathways involved in the recruitment and activation of allergic inflammatory effector cells associated with coronary artery disease, which may reveal new important therapeutic targets (Niccoli et al., 2018) for *de novo* or refractory ISR, thereby reducing the occurrence of this clinical event and the associated risk of long-term cardiovascular events.
